# The Impact of Online Health Anxiety on Maternal Health Behaviors in Cyberchondriac Mothers: A Narrative Review

**DOI:** 10.7759/cureus.105192

**Published:** 2026-03-13

**Authors:** Sobhna Pradhan, Disha Sinha, Aditi Das

**Affiliations:** 1 Obstetric and Gynaecological Nursing, Sri Sri University, Bhubaneswar, IND; 2 Psychiatric Nursing, Sri Sri University, Cuttack, IND; 3 Obstetric and Gynaecological Nursing, Kalinga Institute of Nursing Sciences, Kalinga Institute of Industrial Technology, Deemed to be University (KIIT-DU), Bhubaneswar, IND

**Keywords:** cyberchondria, digital health, maternal health, online health anxiety, perinatal mental health, pregnancy

## Abstract

The growing reliance on internet health information during pregnancy presents two distinct aspects: emotional vulnerability and empowerment. This narrative review examines the impact of cyberchondria on maternal wellness habits and psychological well-being. Cyberchondria is defined by extensive and anxiety-driven health-related web searching. Even though internet platforms can help people make well-informed decisions, excessive and uncontrolled research frequently increases worry, uncertainty, and mistrust of medical providers. Women with cyberchondria during pregnancy and after giving birth may exhibit compulsive symptom-checking, emotional discomfort, weakened bonds, and less confidence as parents. These actions have been linked to higher healthcare use, maternal exhaustion, and possible effects on an infant's development. Social media impact, poor access to treatment, dread of delivery, and insufficient eHealth literacy are all contributing issues. The review emphasizes the need for crucial digital literacy skills development, evidence-based digital therapies, and early detection. The incorporation of digital behavioral health tools into standard prenatal care and inclusive design for marginalized communities is prioritized. Healthcare providers can better support mothers' emotional resilience, enhance caring behaviors, and encourage better outcomes for mothers and their children by addressing internet health anxiety in perinatal situations.

## Introduction and background

The way individuals engage with health information has evolved as a result of the digital revolution. Many people increasingly rely on online platforms to monitor symptoms, get medical advice, and manage their health because of the widespread use of smartphones and simple access to the internet. Convenience has come with this change, but there are also new difficulties, particularly at delicate life phases like pregnancy [1].

Physical changes and mental uncertainties are common throughout pregnancy. It is normal for expecting moms to be filled with worries, questions, and a strong desire to protect their unborn child. Many people use the internet to get reassurance. Digital technologies have become a commonplace companion throughout pregnancy, from reading birth tales and parenting ideas to studying symptoms and fetal development. Because it gives them immediate answers and a feeling of community, this access frequently strengthens women. However, excessive or unregulated internet searching can cause uncertainty, anxiety, and needless concern [2].

Cyberchondria is one such pattern of behavior, which is the propensity to often look up information concerning health online in ways that make anxiety worse rather than better [1]. Pregnant and postpartum women are becoming more aware of this problem. Mothers who suffer from cyberchondria typically feel pressured to monitor their symptoms, compare their experiences to those of others, and fret about infrequent issues [3]. They might have felt more nervous and overwhelmed rather than comforted. Fear of childbirth, mistrust of medical institutions, or emotional fragility during the postpartum period can all exacerbate this trend [4].

Instead of feeling comfortable, they may have felt more anxious and overwhelmed. This tendency may be made worse by postpartum mental fragility, mistrust of medical organizations, or fear of delivery [5]. Although many people found this useful, a sizable percentage acknowledged that it occasionally caused more worry or uncertainty [6]. During pregnancy, primigravida women and those with low digital health literacy having little access to healthcare frequently go to the internet for comfort and support. It becomes the primary information source for many people. However, people might easily misinterpret the information if they do not know how to evaluate it. Increased anxiety and maladaptive coping mechanisms may result from this [7].

Mothers' ideas and feelings might be subtly influenced over time by exposure to perplexing or concerning health information. When common pregnancy symptoms are misconstrued as major problems, what starts as a straightforward quest for confidence can turn into ongoing anxiety and mental stress [8]. Anxiety's mental repercussions frequently manifest first, leading to weariness, psychological anguish, and even melancholy. Even in the absence of a real health issue, mothers may suffer emotional detachment, exhaustion, lack of appetite, or sleep difficulties [9]. Some people could feel alone and unable to express their worries. These emotional strains can affect the mother's overall health, as well as her child's emotional and developmental environment [10].

Maternal behaviors, including attending prenatal checkups, selecting feeding techniques, and adhering to the baby's requirements, are frequently impacted by emotional shifts. They can also affect the general atmosphere of the family and lower parenting confidence. Mothers who suffer from cyberchondria frequently excessively compare medical views, browse several websites, and stress about small symptoms [11]. They may wind up feeling more confused, overburdened, and less secure in their decisions rather than gaining clarity.

This narrative review aims to comprehend how women's attitudes, feelings, and behaviors throughout pregnancy and after delivery are influenced by online health worry, specifically cyberchondria. It attempts to draw attention to the behavioral and psychological trends seen in women who are digitally concerned and investigate how these trends could impact their general well-being and experiences as mothers.

## Review

Search methodology

In order to find pertinent material on the effects of cyberchondria and online health anxiety on maternal health behaviors, this narrative review used a thorough and methodical methodology. Web of Science and PubMed, two large electronic databases, were searched using the following key phrases and their combinations: ("maternal health behavior" OR "pregnancy health behavior" OR "maternal health") AND ("online health anxiety" OR "cyberchondria" OR " medical-related internet anxiety"). To improve and narrow search results, Boolean operators were used.

Studies on pregnant and postpartum women that looked at the behavioral, emotional, and psychological effects of extensive online health information searching were included in the review. Clinical trials, qualitative analysis, observational research, and reviews conducted in English between 2010 and 2025 were all considered eligible studies. A total of 36 publications that offer comprehensive perspectives into how online anxiety about health affects maternal health behaviors, mental well-being, and mother-child relationships during the perinatal period were chosen after being screened for quality, methodological rigor, and relevance.

Cyberchondriac mother: Relationship between online health anxiety and maternal health

Because of its strong correlation with pregnancy-related health concerns, the phenomenon of cyberchondria, which is defined as excessive and obsessive online searches for health-related information, has attracted more attention in maternal health research. Women are prone to increased worries about their own well-being and the well-being of the fetus throughout pregnancy, which is a crucial time of increased emotional sensitivity. Due to this susceptibility and the widespread availability of the internet, pregnant women are increasingly engaging in cyberchondriac behaviors [12]. A woman who is cyberchondriac frequently searches online for health information, which might increase her concern about her own and her children's health. Maternal caring may become more vigilant and exhibit overprotective behaviors as a result of this elevated health-related concern. Excessive medical visits and interventions are frequently the outcome of such anxiety-driven behaviors [13].

Research has repeatedly shown that one of the main mechanisms behind cyberchondria is worry. In particular, increased health concern encourages compulsive and unrestrained internet information searching, which paradoxically exacerbates misery by exposing women to conflicting or unclear health information, thereby sustaining a cycle of anxiety and confusion [2,3]. Interestingly, pregnant women who have obsessive-compulsive behaviors and a higher intolerance for uncertainty are more likely to develop cyberchondria [3,6].

This issue is exacerbated by pregnant women's overreliance on internet resources, many of which lack proper validation, leading to increased worry and misconceptions [14]. Adding to this dynamic, women who lack eHealth literacy are less able to evaluate online information critically, which makes them more vulnerable to misunderstandings and heightened health anxieties. Despite sharing similarities with health anxiety, cyberchondria is conceptually different and is linked to higher levels of functional impairment, higher healthcare usage, and a lower quality of life [15]. Additionally, these worries are exacerbated, and the landscape of maternal mental health is complicated by the presence of online social media websites, which frequently spread alarmist or glorified maternal narratives [9,10]. The significant psychological impact of cyberchondria is further demonstrated by qualitative studies, which show that new moms typically characterize their online medical pursuits as neurotic as well as emotionally burdensome [16].

Impact on maternal health behavior

Digital and online treatments that target psychological and physiological factors during pregnancy and the postpartum period have an impact on mothers' health behaviors. Internet use by pregnant women during pregnancy influences their anxiety about giving birth, which can change physiological stress reactions, such as activity in the autonomic nervous system and hormone changes that affect labor and delivery [17]. The need for therapies to assist healthy maternal adaptation is highlighted by the heightened psychological strain and maternal burnout experienced by postpartum moms who display obsessive and compulsive behaviors combined with raised cyberchondria levels [18]. Underprivileged groups benefit from online therapies that lower prenatal mood and anxiety problems, which are directly related to immunological and cardiovascular system dysregulation [12]. Using virtual reality (VR) during non-stress assessments lowers mothers' anxiety, which improves fetal outcomes and physiological stress indicators [19]. By modifying cortisol and other indicators in underserved populations, VR-based digital therapies further reduce maternal stress and improve pregnancy outcomes [16]. By promoting improved sleep, immunological, and hormonal balance, digital healthcare techniques help manage maternal mental health and promote physiological adjustments essential for both mother and fetal well-being [20]. Together, these therapies help to lower physiological stress and promote healthy mother behaviors throughout the perinatal stage.

Psychological and emotional consequences

Cyberchondria, or the excessive and continuous online search for medical information that ironically creates health worries, has major emotional and psychological consequences for pregnant women [21]. Internet use by expectant parents is common, but for others, this information-seeking behavior becomes a destructive cycle that is made worse by anxiety [1,4].

One of the primary psychological repercussions of cyberchondria is the mutually reinforcing relationship between it and anxiety. Health issues are a significant predictor and associated risk factors for the development of cyberchondria in people in general, and in pregnant women specifically [22]. One research claims that this is a "can't endure, won't stop" phenomenon where pregnant women turn to the internet for solace from their initial fears [1]. However, instead of easing their suffering, the frequently conflicting, comprehensive, and worrisome nature of online health content feeds a vicious cycle [8]. Instead of reducing worries, this compulsive searching lowers quality of life and produces significant psychological injury [11].

The distinct emotional repercussions of cyberchondria have a direct influence on pregnancy. Tokophobia, or a noticeably enhanced fear of giving birth, is one of the most prominent. Research shows a strong link between this kind of internet use and a greater fear of labor and delivery since the search results usually highlight the most dire possibilities and likely issues [23].

Additionally, there is a strong association between increased internet involvement and a considerable spike in prenatal anxiety [24]. Instead of feeling empowered, women with cyberchondria often worry more about their health and the health of their fetus. Women could use online resources more frequently, which further distances them from professional medical guidance and makes their psychological distress worse. When there is already skepticism in the healthcare system, this is exacerbated [6].

Cyberchondria, which extends beyond pregnancy into the postpartum period, starts with a health problem or an unsettling thought that compels you to search online for solutions. Instead of providing comfort, pregnant women are sometimes given information that is worrying or conflicting [2,7]. This sets up a strong psychological loop where she feels more compelled to look the more she explores and the more concerned she becomes. This exacerbates her anxiety rather than allays it [25].

Chronic anxiety and obsessions formed during pregnancy are linked to a higher likelihood of burnout among parents and obsessive-compulsive behaviors related to postpartum baby care [26]. Being constantly worried and on high alert is emotionally draining and can lead to postpartum depression. Furthermore, the internet and the wider digital environment might negatively affect maternal relationships and independently make mothers feel more anxious [9]. Ultimately, searching for health information online proves to be a pointless and emotionally draining endeavor, transforming a moment of anxiety into a time of extreme psychological distress [27].

Implications for maternal and child health

Long-Term Impact on Pregnancy Outcomes

Pregnancy is a period of significant physical and emotional change, during which many women seek health information online. Frequent online searching may increase concerns about maternal and fetal health, contributing to pregnancy-related cyberchondria and heightened health anxiety [1]. This anxiety can influence healthcare-seeking behavior, leading to repeated consultations and requests for additional monitoring [28]. Excessive internet use has also been linked with fear of childbirth, further increasing stress during pregnancy [14]. Consequently, persistent worry may increase healthcare utilization, including unnecessary consultations and repeated diagnostic testing, placing additional burden on maternity services.

When internet information disagrees with expert advice, it can delay appropriate decision-making or lead to misunderstanding. Some women express feeling obligated to double-check medical advice digitally before acting [7]. Unverified internet information has occasionally been connected to a rise in mistrust of medical professionals, which makes pregnancy even more stressful [4]. Over time, all of these impacts may have an impact on the general standard of prenatal care as well as the well-being of the mother [8].

Effect on Infant Development and Bonding

Bonding develops through consistent, receptive encounters during the first several months of life. Anxiety that results in frequent internet health searches might cause a woman to focus more on symptom monitoring than on bonding with her child [29]. Overuse of internet health resources might diminish emotional availability, leading to fewer instances of responsive conversation, loving touch, and eye contact [9]. Qualitative research shows that moms with a severe case of cyberchondria frequently have mental distractions during feeding or comforting, which reduces their ability to connect deeply emotionally [10].

Additionally, postpartum research links mother fatigue to compulsive health-related behaviors, which might compromise the standard of early caring [30]. Mothers who are under a lot of stress frequently complain of exhaustion, depression, and trouble keeping up regular caregiving schedules [31]. If this pattern continues, it may influence the infant's emotional control and sense of stability by impacting early relationship experiences [32]. Evidence now available indicates that persistent maternal worry at this critical time can have long-lasting developmental effects, even if additional longitudinal studies are required [33].

Family Dynamics and Partner Support

Support from family members is essential for assisting expectant women in managing their anxiety. Relationship conflict can occasionally arise from excessive online health searches, particularly if partners feel overburdened by the constant discussion of online results or excluded from decision-making [34]. According to studies, maternal anxiety is much decreased when partners actively participate in prenatal care by going to checkups, assisting with the interpretation of medical information, promoting responsible internet usage, and providing comfort [35].

On the other hand, alarmist or negative partner reactions can exacerbate discomfort and fuel conflict. Involving both parents in digital health literacy programs can enhance collaborative decision-making and minimize needless anxiety [36]. Together, couples who learn to assess internet material are more equipped to weed out dubious sources, keep faith in medical professionals, and concentrate on making healthy birth and parenting preparations [25]. Enhancing this cooperative strategy helps safeguard the mother's health and provide a more positive emotional atmosphere for the baby (Table 1).

**Table 1:** Features of reviewed articles VR: virtual reality, OCD: obsessive-compulsive disorder, ICTs: information and communication technologies

Author	Year	Key findings	Conclusion
Antoniou et al. [1]	2014	Maternal pre-pregnancy weight was associated with externalizing behavior in children.	Maternal weight before pregnancy may influence child behavior.
Carona et al. [2]	2023	Web-based intervention "Be a Mom" reduced postpartum depression risks.	Digital interventions can be effective in preventing postpartum depression.
Carrasco et al. [3]	2024	Protocol to test VR therapeutic to reduce maternal stress in Black and Latina mothers.	VR-based tools have potential for maternal mental health support.
Coglianese et al. [4]	2020	Online health information-seeking increased anxiety in hospitalized pregnant women.	Caution is needed when using online health information during pregnancy.
Giacometti et al. [5]	2024	Pregnant women use the internet for prenatal information, but vary in trust and understanding.	Digital literacy is crucial in prenatal internet use.
Guille et al. [6]	2024	Digital healthcare can improve maternal mental health management.	Integration of digital tools is promising for maternal care.
Henton and Swanson [7]	2023	Online social support promotes psychological well-being in new mothers.	Digital peer support is beneficial for maternal mental health.
Liblub et al. [8]	2024	Peer support and mobile health aid for perinatal mental health.	Combining peer and mobile health approaches shows promise.
Nadeem et al. [9]	2022	Health anxiety strongly linked to cyberchondria, mediated by metacognitive beliefs.	Targeting metacognitive beliefs may reduce cyberchondria.
Rathbone and Prescott [10]	2019	New parents felt anxious due to overwhelming online health info.	Better digital guidance can help reduce parental anxiety.
Šoštarić et al. [11]	2023	Risk factors and triggers identified for cyberchondria in pregnant women.	Monitoring anxiety levels may help manage cyberchondria.
Waring et al. [12]	2024	Online platforms were used for maternal mental health engagement and education.	Digital outreach can successfully engage perinatal populations.
Zhou et al. [13]	2021	Fear of childbirth was linked to several psychosocial risk factors.	Psychosocial support can mitigate childbirth-related fear.
Suarez-Lledo and Mejova [14]	2022	Online health campaigns led to behavior change.	Digital awareness campaigns can influence health behaviors.
Alvarez et al. [15]	2022	Mothers reconstruct identity online after terminating pregnancies for health reasons.	Online storytelling helps process traumatic maternal experiences.
Baradwan et al. [16]	2025	VR reduced maternal anxiety and improved satisfaction during non-stress tests.	VR is an effective supportive tool in prenatal care.
Berger et al. [17]	2024	X (Twitter) users shared distress and coping related to hyperemesis gravidarum.	Social media offers insight into maternal illness experiences.
Canfield and Canada [18]	2023	Online interventions reduced perinatal mood/anxiety disorders in underserved populations.	eHealth is effective for vulnerable groups.
Chi et al. [19]	2020	Online information-seeking had offline health consequences.	Online behavior impacts offline health decisions.
Gong and Bharj [20]	2022	Chinese migrant women used digital resources to navigate UK maternity care.	Culturally tailored digital tools can support migrant maternal care.
Gulec Satir and Bakir [21]	2024	Cyberchondria positively correlated with health anxiety in pregnant women.	Addressing health anxiety may help reduce cyberchondria.
Kizilirmak and Calpbinici [22]	2022	Internet use about childbirth increased fear of childbirth.	Unfiltered internet information may exacerbate fears.
Kurcer et al. [23]	2022	COVID-19 heightened health anxiety and cyberchondria in students.	Pandemics amplify digital health anxieties.
Li et al. [24]	2025	Maternal sleep and mental health linked; family support moderated effects.	Family function plays a protective role in maternal mental health.
Mathes et al. [25]	2018	Cyberchondria overlapped with health anxiety lowered quality of life.	Cyberchondria negatively affects well-being.
Monteiro et al. [26]	2022	Web-based maternal mental health intervention was cost-effective.	Digital care is cost-efficient for low-risk mothers.
Moore et al. [27]	2020	Online forums reduced stigma and supported women with maternal mental illness.	Forums are a valuable stigma-reducing resource.
Rajput et al. [28]	2024	Social media affects maternal attachment and anxiety.	Excessive social media use may hinder maternal bonding.
Gupte [29]	2024	Found a direct relationship between cyberchondria and health anxiety.	Cyberchondria intervention should target anxiety.
Şatır [30]	2023	Expectant fathers with higher anxiety searched more online.	Men's digital health behavior reflects emotional states.
Šoštarić et al. [31]	2024	Anxiety predicted higher cyberchondria in pregnant women.	Anxiety reduction may help manage excessive online searching.
Soyler et al. [32]	2023	Health anxiety and mistrust in healthcare increased cyberchondria.	Trust in systems can reduce harmful online behavior.
Starcevic and Berle [33]	2013	Excessive health-related internet use defined as cyberchondria.	Conceptual clarity is key for intervention.
Uslu-Sahan and Purtul [34]	2023	Low eHealth literacy and high health anxiety predicted cyberchondria in women.	Improving digital literacy may lower cyberchondria.
Tiryaki et al. [35]	2025	Cyberchondria and OCD linked to maternal burnout postpartum.	Mental health support is essential after childbirth.
Vaidean and Achim [36]	2022	ICTs improved health only up to a point; overuse reduced benefits.	Balance is crucial in digital health use.

Recommendation

A multimodal strategy based on medical practice and technological innovation is needed to address the impact of internet-based health anxiety on maternal health habits. Assessments for excessive internet health information-seeking and health-related anxiety should be part of routine maternity healthcare. Early detection of these trends might provide prompt treatments, minimizing possible adverse effects on the mental health and decision-making of mothers. Prenatal and postnatal care should incorporate evidence-based digital resources created especially for the mental health of mothers. Cognitive-behavioral support, mindfulness-based techniques, and psychoeducation are all components of web-based and mobile therapy that have shown promise in lowering anxiety and fostering emotional resilience. Particularly for moms who have limited access to in-person mental health treatments, these platforms provide scaled and easily accessible solutions.

Enhancing digital health literacy is still of utmost importance. Giving moms the ability to assess internet material critically can help reduce the rise in anxiety that is frequently linked to unfiltered or contradictory content. The safe and knowledgeable use of electronic assets can be improved by structured instructional programs that are incorporated into maternity care pathways. Networks of social support, especially those made possible by internet platforms, provide significant psychological advantages. These communities have the potential to normalize emotional difficulties, lessen feelings of loneliness, and promote peer support when properly controlled. To make sure that these platforms do not unintentionally promote false information or unhealthy coping mechanisms, healthcare experts' advice is crucial.

Ensuring equitable maternal health outcomes requires digital health interventions that address socioeconomic, linguistic, and cultural differences. Access to reliable online health information remains challenging for underserved populations, highlighting the need for inclusive and accessible digital platforms. Further research, particularly longitudinal studies, is needed for a better understanding of the causal relationships between maternal behaviors and cyberchondria. In addition, strategic policy development and infrastructure investments are essential for the safe and effective integration of digital mental health services into routine maternity care.

## Conclusions

Cyberchondria is an increasing threat to mothers' psychological well-being during pregnancy and after giving birth, which is fueled by excessive and concerned internet health-seeking. Although the internet makes it simple to get information and assistance, if it is not utilized carefully, it may also cause confusion, increased worry, and abnormal behaviors in mothers. Prenatal decision-making may suffer, unneeded healthcare utilization may rise, family ties may be strained, and early bonding with the unborn child may be weakened as a result of this trend. Healthcare systems need to incorporate regular screening for virtual health anxiety, enhance digital health literacy, and offer evidence-based digital solutions that promote emotional resilience in order to address this. Maternal well-being may be further supported through online communities, culturally sensitive initiatives, and greater partner involvement in digital health education. When used appropriately, digital tools can empower mothers with reliable information, reduce anxiety, and promote improved health outcomes for both mothers and their children.
